# The Metallothionein Gene, *TaMT3*, from *Tamarix androssowii* Confers Cd^2+^ Tolerance in Tobacco

**DOI:** 10.3390/ijms150610398

**Published:** 2014-06-10

**Authors:** Boru Zhou, Wenjing Yao, Shengji Wang, Xinwang Wang, Tingbo Jiang

**Affiliations:** 1 State Key Laboratory of Tree Genetics and Breeding, Northeast Forestry University, Harbin 150040, China; E-Mails: boruzhou@yahoo.com (B.Z.); wenjingyao20140408@gmail.com (W.Y.); shengjinefu@gmail.com (S.W.); wlywing@yahoo.com (X.W.); 2 Texas A&M AgriLife Research and Extension Center, Texas A&M System, Dallas, TX 75252, USA

**Keywords:** cadmium, genetic transformation, metallothionein, *Tamarix androssowii*, tobacco

## Abstract

Cadmium (Cd) is a nonessential microelement and low concentration Cd^2+^ has strong toxicity to plant growth. Plant metallothioneins, a class of low molecular, cystein(Cys)-rich and heavy-metal binding proteins, play an important role in both metal chaperoning and scavenging of reactive oxygen species (ROS) with their large number of cysteine residues and therefore, protect plants from oxidative damage. In this study, a metallothionein gene, *TaMT3*, isolated from *Tamarix androssowii* was transformed into tobacco (*Nicotiana tobacum*) through *Agrobacterium*-mediated leaf disc method, and correctly expressed under the control of 35S promoter. Under Cd^2+^ stress, the transgenic tobacco showed significant increases of superoxide dismutase (SOD) activity and chlorophyll concentration, but decreases of peroxidase (POD) activity and malondialdehyde (MDA) accumulation when compared to the non-transgenic tobacco. Vigorous growth of transgenic tobacco was observed at the early development stages, resulting in plant height and fresh weight were significantly larger than those of the non-transgenic tobacco under Cd^2+^ stress. These results demonstrated that the expression of the exogenous *TaMT3* gene increased the ability of ROS cleaning-up, indicating a stronger tolerance to Cd^2+^ stress.

## 1. Introduction

Cadmium (Cd) is a kind of unnecessary microelement and heavily toxic to plant growth and development. Low concentration of Cd^2+^ could cause inhibitive influence on plant cell growth, oxidative phosphorylation, photosynthesis, and therefore damage the nucleolus and membrane ATPase activity of plant cells. Additionally, Cd^2+^ contamination leads to the degeneration of soil fertility, reduces the crop production and quality, deteriorates water resources, and eventually jeopardizes human health directly through the food chains [1,2,3,4]. Currently, the remediation of heavy-metal contaminated soil is a worldwide challenge because the metal ions are hard to remove [5]. The traditional remediation methodologies mainly resort to some physical or chemical techniques: thus, they could not be applied to large areas of contaminated soils because of the high costs. An alternative approach, phytoremediation could decrease the costs and be applied to large areas [6]. The core work of phytoremediation is the development of plant varieties resistant to heavy metals. Genetic transformation of important genes was an important approach to plant breeding.

Metallothioneins (MTs) are defined as low-molecular Cys-rich proteins that bind heavy metals in plant and animal cells. MTs are widely distributed in eukaryotic and prokaryotic species and have been demonstrated as playing an important role on the detoxification of heavy metals in animals and fungi [7]. It was found that MTs generally impact the species tolerance to heavy metals at the RNA level [8,9], suggesting they have metal binding ability and homeostasis. The genes encoding the MTs have been identified and cloned from many plant species such as wheat [10,11], Arabidopsis [8], soybean [12], tomato [13], and fescue [14]. Three major classes of MTs have been found in plants [7,8]. Class-I MT-like proteins are characterized by two Cys-rich domains separated by a central Cys-free spacer; class-II MTs are represented by the wheat Ec protein, lacking the internal spacer [11]; class-III MTs include diverse glutathione-derived peptides as phytochelatins [15]. Many stimuli such as metal ions, mechanical damage, virus infection, and heat shock can induce the expression of *Metallothionein* (*MT*) genes and accumulation of MTs in plants [16]. In addition, the transgenic plant containing an exogenous animal *MT* gene presented a high resistance to Cd^2+^, Pb^2+^, Zn^2+^ and other heavy-metal ions [17,18,19].

*Tamarix androssowii*, a plant growing in desert areas, has strong tolerance to drought, salt, and alkaline under the natural environments. Our previous studies of gene expression patterns revealed that the most abundant Expressed Sequence Tag (EST) found in *Tamarix androssowii* under NaHCO_3_ stress encoded metallothionein [20]. In this study, the gene *TaMT3* encoding *Tamarix* metallothionein was introduced into tobacco genomes by the *Agrobacterium*-mediated transformation method. In order to examine the anti-heavy-metal role of the gene *TaMT3* and to explore its potential for further application in plant breeding for wide environmental adaptation, the expression in transgenic tobacco was characterized by measuring the physiological traits of transgenic plants under Cd^2+^ stress conditions.

## 2. Results

### 2.1. Isolation of cDNA Encoding Type-II Metallothionein

**Figure 1:**
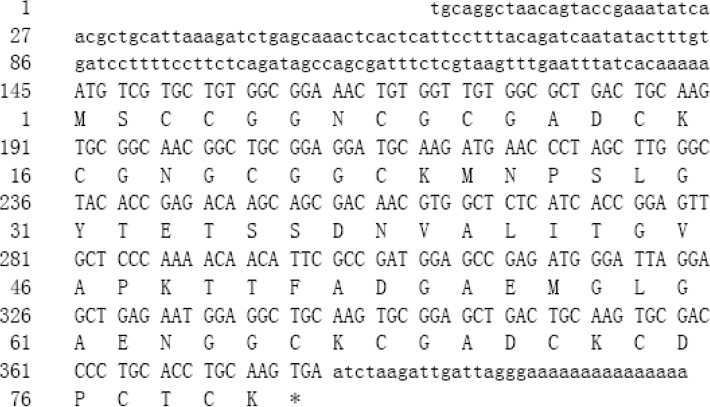
Nucleotide and deduced amino acid sequences of the *TaMT3* cDNA. Lower-case letters indicate the 5'- and 3'-UTRs and upper-case letters the open reading frame.

The complete sequence of metallothothionein cDNA designated as *TaMT3* was obtained using 5'/3' RACE Kit. The sequence of *TaMT3* cDNA consisted of 420 nucleotides, encoded 80 amino acid residues in an open reading frame (ORF), that is preceded by a 5' untranslated region (UTR) of 144 nucleotides and followed by a 3'-UTR of 33 nucleotides (Figure 1). The ORF started in a favorable context for translation initiation. The protein BLAST results showed that the molecular properties of *TaMT3* were very similar to those of major known plant metallothioneins. For example, *TaMT3* had 75% similarity with MT from *Arachis hypogaea* (ABA08415), 66% with MT from *Ilex paraguariensis* (AFP93964) and 65% with MT from *Limonium bicolor* (ABL10085). Based on its Cys spacing and Cys residues in each of the two domains, *TaMT3* was belonged to type-II of plant class-I MTs.

### 2.2. Expression of TaMT3 Gene in Response to CdCl_2_ Stress

**Figure 2:**
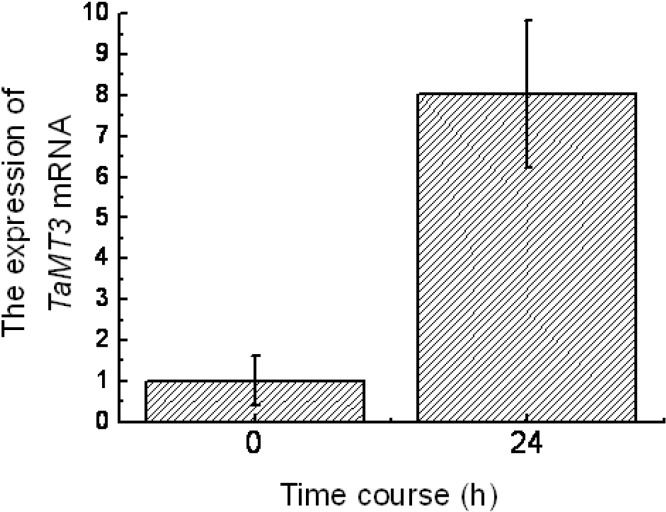
The expression of *TaMT3* mRNA detected by reverse transcription quantitative polymerase chain reaction (RT-qPCR).

The young twigs of *Tamarix androssowii* plants were cultured in water for two weeks and placed in CdCl_2_ solution for 24 h. The effects of cadmium on relative abundance of *TaMT3* mRNA were analyzed using reverse transcription quantitative polymerase chain reaction (RT-qPCR). The relative abundance of *TaMT3* mRNA was 8-fold higher after 24 h of Cd^2+^ stress than that of the control (0 h) (Figure 2), indicating that the expression of *TaMT3* gene was induced by Cd^2+^ stress.

### 2.3. Expression of TaMT3 Gene in Transgenic Tobacco Plants

**Figure 3:**
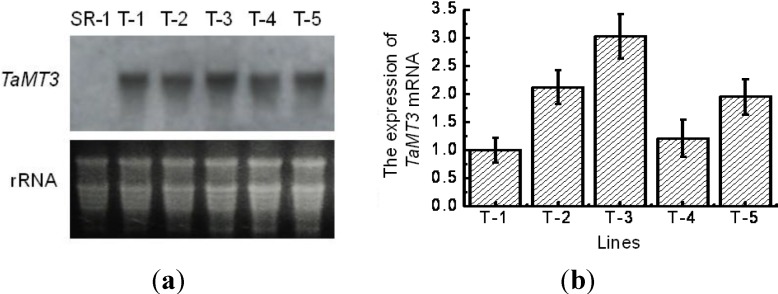
The expression analysis of *TaMT3* gene in transgenic tobacco by northern blot and reverse transcription quantitative polymerase chain reaction (RT-qPCR) (**a**) northern blot analysis; and (**b**) RT-qPCR analysis using specific primers. SR-1, non-transformant; T-1 to T-5, transgenic lines.

After polymerase chain reaction (PCR) analysis, five putative transgenic tobacco lines were subjected to Northern blotting analysis in order to examine the exogenous gene expression on transcriptional level. The results showed that all five transgenic lines showed the hybridization signals except the control plant (SR-1), indicating the exogenous gene expressed in tobacco leaves (Figure 3). Of the five transgenic tobacco lines, T-2, T-3 and T-5 showed stronger signals and T-1 and T-4 relatively lower signals, indicating that *TaMT3* gene has different expression levels in different transgenic tobacco plants.

### 2.4. Responses to Cd^2+^ Resistance in Transgenic Tobacco

**Figure 4:**
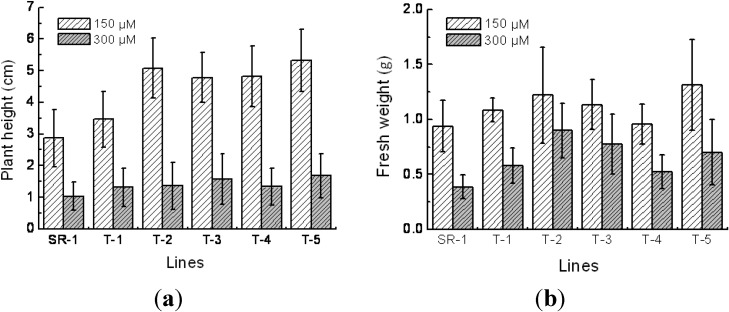
Comparison of plant height (**a**) and fresh weight (**b**) between transgenic and non-transgenic tobacco plants. SR-1, non-transgenic tobacco; T-1 to T-5, transgenic tobacco. Mean values and standard deviations were calculated based on six replicates.

For understanding of differences of morphological and physiological character between transgenic tobacco and wild type in normal conditions, the chlorophyll and malondialdehyde (MDA) content, plant height, fresh weight, superoxide dismutase (SOD) and peroxidase (POD) activity were measured. Results indicated that there was no morphological and physiological change between transgenic plants and wild type in normal conditions (data not shown). After 2-month growth on Murashige and Skoog (MS) medium supplemented with 150 and 300 μM of Cd^2+^, the plant height and fresh weight of the transgenic and nontransgenic lines were measured. The results showed that the growth performance of both transgenic and nontransgenic plants was significantly inhibited on 300 µM of Cd^2+^ medium when compared to on 150 μM of Cd^2+^ medium. However, the transgenic lines had a larger plant height and fresh weight than the nontransgenic plants under both Cd^2+^ stress conditions (Figure 4a,b). In the 150 μM of Cd^2+^ condition, the plant height of the transgenic lines were 21%–87% higher than the nontransgenic plants significantly at *p* < 0.05. Similarly, in the 300 μM of Cd^2+^ stress condition, the fresh weight of the transgenic plants were 38%–133% higher than the nontransgenic plants significantly at *p* < 0.05. In addition, there was no significant linear relationship between the increase of the plant height and fresh weight of the transgenic lines and the transcription amount of exogenous genes under the Cd^2+^ stress conditions.

### 2.5. Physiological Character of Transgenic Tobacco

**Figure 5:**
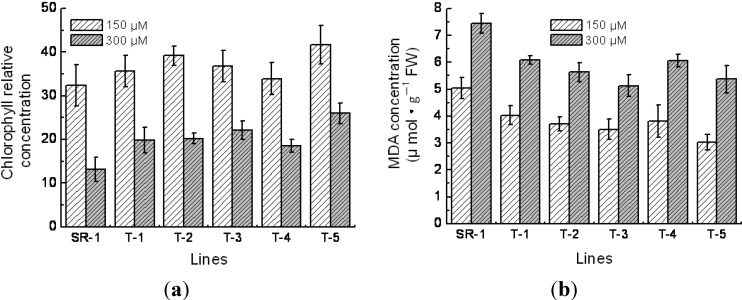
Comparison of chlorophyll (**a**) and malondialdehyde (MDA) (**b**) concentration between transgenic and non-transgenic tobacco lines. SR-1, non-transgenic tobacco; T-1 to T-5, transgenic tobacco. Mean values and standard deviations were calculated based on the six replicates.

Chlorophyll is an indispensable constituent of chloroplast and is vital important in photosynthesis. Hence, in various circumstances, its content can be used as an indicator to reflect the photosynthesis ability and carbohydrates assimilation potentiality. In this study, we found that the average chlorophyll content in transgenic tobacco was 60% higher when grown under 150 μM Cd^2+^ conditions than grown in 300 μM of Cd^2+^ condition (Figure 5a). Meanwhile, in the 150 μM Cd^2+^ concentration medium, the average chlorophyll content of transgenic lines was 0%–18% at no significant levels, and on the 300 μM Cd^2+^ concentration was 40%–113% higher than the nontransgenic plants at the significant levels (*p* < 0.01). These results indicated that the exogenous expression of *TaMT3* gene prevents the degradation of chlorophyll when suffering Cd^2+^ stress.

Under drought and salt stress conditions, the active oxygen species (ROS) in plant cells can induce the oxidation of unsaturated fatty acid, thus damage the membrane system [21]. One of the end products of lipid overoxidation is MDA, and its content is often used as an indicator of cell membrane lipid overoxidation and the degree of cell membrane injury [22]. In the present study, the MDA content of transgenic lines grown on each of the Cd^2+^ concentration medium were 20%–40% lower than that of the non-transformants at the significant levels (*p* < 0.01) (Figure 5b), indicating that the over-expression of *TaMT3* gene reduced the lipid overoxidation rate in transgenic plants.

**Figure 6:**
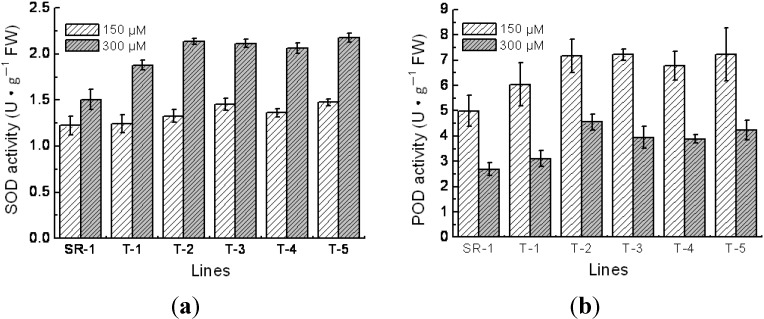
Comparison of SOD (**a**) and POD (**b**) activity among transgenic and non-transgenic tobacco lines. SR-1, non-transgenic tobacco; T-1 to T-5, transgenic tobacco. Mean values and standard deviations were calculated based on the six replicates.

SOD and POD can clean the oxygen free radicals, maintain the metabolic balance of active oxygen species, and consequently prevent the overoxide of membrane lipid and protect the cell membrane system [22,23]. Comparing the SOD and POD activities in each line, it revealed that the SOD activity was increased but the POD activity was decreased, when the Cd^2+^ concentration was increased. Furthermore, under the 150 and 300 μM Cd^2+^ condition, the SOD activity of the transgenic lines were 1.6%–21% and 24%–44% higher than that of the nontransgenic plants, respectively (*p* < 0.01, Figure 6a), and the POD activity of the transgenic lines under the 150 and 300 μM Cd^2+^ condition were 15%–69% and 21%–45% higher than that of the nontransgenic plants, respectively (*p* < 0.01, Figure 6b). These results demonstrated that the over-expression of *TaMT3* gene elevated the SOD and POD activities in the cells.

## 3. Discussion

Although many recent studies have revealed the roles of MTs in plants in response to diverse metal stresses, there is still much more information needed. The larger diversity in the metal-binding regions of plant MTs suggests that they have ability to bind a greater range of metals than their animal counterparts and, consequently, a greater range of function [24,25]. The *TaMT* gene from *Tamarix androssowii* leaves was determined as type-II MT based on the protein sequence structure but it was different from other type-II MTs in plants (e.g., *AsMT2a* in Zhang *et al*.; *AsMT2b* in Zhang *et al*.) [26]. It has been found that the expression of plant MTs is tissue-specific and type-II MT mRNA is abundant in leaves [27]. The *TaMT* gene overexpressed under the control of 35S promoter in transgenic tobacco plants, resulting not only in significant increase of SOD and POD activities, but also in an increase of plant height and fresh weight when suffered 150 to 300 μM Cd^2+^ stress compared to non-transgenic tobacco plants. Thus, the *TaMT* gene in *Amarix androssowii* plays an important role in accumulation of Cd^2+^ ions in the tobacco plant cells.

Reactive oxygen species (ROS) are chemically reactive molecules containing oxygen. Effects of ROS on cell metabolism are well documented in a variety of species. They are formed as a natural byproduct of the normal metabolism of oxygen and have important roles in cell signaling and homeostasis [28]. According to the Free-radical Theory, oxidative damage initiated by ROS is a major contributor to the injury of cells [22]. When plants are exposed to stress, the stress induces the ROS that hurts the cell membrane system by overoxidating the fragile phospholipins in membrane and fatty acid in cytoplasm and changing the dimensional conformation and permeability of membrane proteins, thus, ultimately causing the death of cells [29,30]. Cadmium stress can also induce the production of oxygen free radicals and peroxide [16,31,32], which injure the photosynthetic apparatus and thus affect the photosynthesis [33,34]. The cadmium itself can cause cell damage through different pathways, such as interfering the water metabolism, decreasing the chlorophyll content, changing the permeability of cell membrane, reacting with hydrosulfide group of many proteins, contending for active site with other substrate, competing with other necessary nutrient elements, and changing DNA conformation [35]. There are many such antioxidant reductases and compounds like SOD and POD that can clean the ROS in cells. The injury level and stress-resistant ability of plant have a positive correlation activation of SOD and POD [36].

*MT* gene is an important responsive gene in genus *Tamarix* when plants have suffered environmental stress. Through the analysis of the EST library constructed from the salt-treated *Tamarix*, Wang *et al.* [20] found that the most abundant transcripts in the EST library are *MT* genes, which account for 3.5 percent of all the 2455 clones. This result indicated that the accumulation of *MT* played a crucial role on stress resistance in *Tamarix* plants. This study indicated that the plant height, fresh weight and chlorophyll content in transgenic tobacco plants decreased when Cd^2+^ concentration increased, in agreement with the previous studies [37]. However, the growths of transgenic lines were much better than the non-transgenic plants. In addition, the over-expression of the *TaMT3* gene elevated the SOD activity but decreased the MDA content, an indicator of oxidation of lipids in cells. These results demonstrated that the over-expression of the *TaMT3* gene has a protective role in photosynthesis apparatus and antioxidation system in cells, because of the reduction of hydrosulfide groups or the heavy-metal binding function of the *TaMT3* gene.

This study found that the young leaves of transgenic tobacco plants grown on MS medium supplemented with Cd^2+^ were white at the beginning, and then their color turned into green in two weeks. However, the leaves of the non-transgenic tobacco plants grown on the same medium did not change to green. This morphological difference directly demonstrated that the protective role of the *TaMT3* gene on the plant growth and development. However, there is no significant linear correlation between the level of transcription of exogenous *TaMT3* gene and the anti-Cd^2+^ ability or the physiological traits of transgenic lines, suggesting that the anti-heavy metal physiological machinery is a complex system. In this study, we found that, under 300 μM Cd^2+^ stress conditions, the two transgenic plants (T-2 and T-3) had more than twice the average plant weight of the non-transgenic plants, compared with 1.8 times the plant weight of the non-transgenic plants for the rat MTα [31]. This disparity could be caused by the different adaptive mechanisms of plant and animal to living environment. Given the roles on heavy-binding, we might utilize the *TaMT3* gene to breed heavy metal resistant varieties of crops and forest trees in the future.

## 4. Materials and Methods

### 4.1. Materials

The twig cuttings of *Tamarix androssowii* plants were harvested and kept in containers in water. The containers were placed in a controlled greenhouse conditions of 60%–70% relative humidity, 16 h light/8 h dark, and an average temperature of 25 °C. Two-weeks twigs were then subjected to the following treatments: 150 μM CdCl_2_ for 0 and 24 h. Leaves were collected from at least three seedlings after each treatment in order to have enough samples. The pooled leaf samples were frozen immediately in liquid nitrogen and stored at −70 °C for RNA isolation. Total RNA was extracted using Column Plant RNAout Kit (Tiandz, Beijing, China) and proceeded to cDNA synthesis using a cDNA Synthesis kit (Takara, Dalian, China) according to the manufacturer’s instructions.

One metallothionein EST (accession No. CV792539) [20] containing an opening frame from *Tamarix androssowii* was chosen to design primers (forward primer 5'-GATGCAAGATGAACCCTAGCTTGG-3' and reverse primer 5'-GCACTTGCAGCCTCCATTCTCAGC-3'), and the complete sequence of metallothothionein cDNA designated as *TaMT3* was obtained using 5'/3' RACE Kit (Roche Molecular Biochemicals, Mannheim, Germany), and the proteins assumed from the amino acid sequences were analyzed using the BLAST program at National Center for Biotechnology Information (Bethesda, MD, USA) [38]. The fragment of protein coding segment of the *TaMT3* cDNA was obtained by RT-PCR (Takara, Dalian, China) using a pair of primers (forward primer 5'-CGTCTAGATGTCGTGCTGTGGCGGAAAC-3' and reverse primer 5'-GCGAGCTCCCCTAATCAATCTTAGATTC-3'). The additional XbaI and SacI restriction sites at the 5' and 3' ends of *TaMT3* cDNA allowed directional cloning into the pBI121 binary vector, in place of the XbaI–SacI beta-glucuronidases (GUS) cassette. The Cauliflower mosaic virus (CaMV) 35S promoter/nopalin synthase (NOS) terminator system and kanamycin resistant gene (*NPTII*, *neomycin phosphotransfers II*) were used for these constitutive expression systems. The *TaMT3* cDNA was introduced into the tobacco (*Nicotiana tabacum* L. cv. Petit Havana SR-1) genome by employing the Agrobacteria-mediated transformation method, and transformations were confirmed by PCR analysis. T_2_ homozygous seeds of five transgenic lines from T_1_ generation presenting 3:1 segregation under kanamycin screening were selected for further stress experiments.

### 4.2. Reverse Transcription Quantitative Polymerase Chain Reaction (RT-qPCR) Analysis

Approximately 1 μg of total RNA was reverse-transcribed to cDNA in a 20 μL volume using the PrimeScript™ RT reagent Kit with gDNA Eraser (Takara, Dalian, China). The synthesized cDNA was diluted to 100 μL with sterile water and used as the template for qPCR. To decrease replicated experimental errors for each sample, purified RNA from the three sub-samples were pooled with equal molars. Three technical replications were performed for each sample to assess the reproducibility, and the mean of the three replications was used to calculate relative expression fold changes. The qPCR was performed on a DNA Engine Opticon Continuous Fluorescence Detection System (MJ Research, Waltham, MA, USA). The qPCR was performed using SYBR Premix Ex Taq™ kit (Takara, Dalian, China) according to the manufacturer’s instructions. A total reaction volume of 25 μL containing 1 μL cDNA (equivalent to 100 ng total RNA) and 25 μM of each primer was used in these analyses. Cycling parameters were set up according to the recommendation of qPCR kit. The amplification curve was generated after analyzing the raw data, and the cycle threshold (*C*_t_) value was calculated based on the fluorescence threshold as 0.01. The expression level of *Tamarix* actin amplified with primer pair Actin-F: 5'-CTGGATTGGAGGTTCTATCTTGGC-3' and Actin-R: 5'-CTGTGGACGATTGAAGGACCAGAC-3' was used as an internal control of *Tamarix androssowii*. The expression level of tobacco GAPDH (accession No. M14419) amplified with primer pair GAPDH-F452: 5'-GCACTACCAACTGCCTTGCACCT-3' and GAPDH-R452: 5'-GGCAATTCCAGCCTTGGCATCG-3' was used as an internal control of tobacco. The primers MT-F: TGTCGTGCTGTGGCGGAAAC-3' and MT-R: 5'-CCCTAATCAATCTTAGATTC-3' were used for the analysis of the expression levels of the *TaMT3* mRNA. The relative expression level of target genes in different samples was calculated using 2^−ΔΔ*C*t^ method, defined as: ΔΔ*C*_t_ = (*C*_t-target_ − *C*_t-control_)_2_ − (*C*_t-target_ − *C*_t-control_)_1_ [39].

### 4.3. Northern Blotting Analysis

The plantlets presenting a 3:1 segregation ratio on MS medium containing 100 mg·L^−1^ of kanamycin were used as plant materials for RNA isolation. Total RNA were isolated using TRIzol Reagent (Invitrogen, Carlsbad, CA, USA) and 30 µg of them were denatured at 100 °C for 5 min. After separation on 1% formaldehyde denaturing agarose gel, the RNAs were transferred onto positively charged nylon membrane (Amersham Life Science, Oakville, ON, Canada) according to the manufacturer’s instruction. The probe from *TaMT3* cDNA used for subsequent hybridization was synthesized by PCR DIG probe synthesize kit (Roche, Foster City, CA, USA). The northern blot was performed according to the procedure described by Engler-Blum *et al*. [40].

### 4.4. Characterization of Growth Traits

Seeds of five transgenic tobacco lines were surface-sterilized with sodium hypochlorite and planted onto MS medium containing 100 mg·L^−1^ of kanamycin. The seeds of nontransformant planted to MS medium without kanamycin were used as control. After one-month growth under 26 ± 1 °C, 16 h light/8 h dark condition, the plants were transplanted to individual glass bottles containing MS medium supplement with 150 and 300 µM Cd^2+^, with 6 replications. After 2 months, the plant height and fresh weight were measured.

### 4.5. Measurements of Superoxide Dismutase (SOD), Peroxidase (POD) Activity, Chlorophyll Content and Malondialdehyde (MDA) Content

SOD activity was measured by using nitro blue tetrazolium (NBT) photoreduction method [41], and POD activity was determined by using the guaiacol method [42]. The thiobarbituric acid colouration method was employed to determine MDA content [43], and SPAD-502 portable chlorophyll meter (Minolta Corporation LTD, Osaka, Japan) was used to measure the relative chlorophyll content.

## 5. Conclusions

*TaMT3* gene isolated from *Tamarix androssowii* improved the Cd^2+^ resistance in the transgenic tobacco plants. This improvement was manifested by the elevated plant height and fresh weight under Cd^2+^ stress conditions. In addition, the transgenic tobacco plants showed a higher SOD and POD activity and lower MDA content in cells.
